# Role of new digital technologies and telemedicine in pulmonary rehabilitation: Smart devices in the treatment of chronic respiratory diseases

**DOI:** 10.1007/s00508-021-01930-y

**Published:** 2021-08-30

**Authors:** Monika Fekete, Vince Fazekas-Pongor, Peter Balazs, Stefano Tarantini, Anna N. Nemeth, Janos Tamas Varga

**Affiliations:** 1 Department of Public Health, Faculty of Medicine, Semmelweis University, Budapest, Hungary; 2 Department of Biochemistry and Molecular Biology, University of Oklahoma Health Sciences Center, Oklahoma City, OK USA; 3 Department of Health Promotion Sciences, College of Public Health, University of Oklahoma Health Sciences Center, Oklahoma City, OK USA; 4 Department of Pulmonology, Semmelweis University, Budapest, Hungary

**Keywords:** Respiratory diseases, Pulmonary rehabilitation, High-technology, Smart devices, Telemedicina

## Abstract

**Background:**

Asthma and chronic obstructive pulmonary diseases are conditions characterized by a variable progression. Some individuals experience longer asymptomatic periods while others acute worsening periods and/or exacerbations triggered by symptom multiplication factors. Medications are adjusted to the patients’ respiratory function, self-assessment of health and emerging certain physical changes. A more effective treatment may be applied by real-time data registered during the patient’s everyday life.

**Aim and methods:**

Introducing new modern digital technology in pulmonary rehabilitation (PR) to help tracking the patients’ medication, thus we systematically reviewed the latest publications on telemedicine and pulmonary telerehabilitation.

**Conclusion:**

The use of the latest digital technologies in PR is very exciting and offers great opportunities while treating patients affected by specific conditions. On the one hand, adherence to medication can be improved in patients with chronic respiratory diseases by using these new state of the art devices; on the other hand, digital devices will also be able to monitor various physiological parameters of patients during their usual everyday activities. Data can be stored on a smartphone and shared with the provider. Relying on this information, physicians will be able to tailor medications and dosage to the specific needs of individual patients. Telerehabilitation may be a sustainable solution to the growing burden of chronic respiratory disease worldwide. However, PR must keep its cornerstones, such as education and motivations, which are most successful when conducted in person. Many issues remain to be resolved in the future, e.g. cybersecurity while using smart devices since they offer unique opportunities for PR.

## Introduction

Pulmonologists struggle with chronic respiratory conditions every day. Asthma and chronic obstructive pulmonary disease (COPD) are among the most common ailments in our modern world, and their numbers are continuously increasing [1]. The World Health Organization (WHO) estimates that currently 235 million people suffer from asthma, approximately 300,000 new patients are diagnosed every year and nearly 250,000 people die prematurely each year due to the illness [2]. The other very common condition COPD affects 64 million people worldwide. According to WHO predictions, by 2030 the disease will become the third most common cause of death in the world due to increased air pollution and tobacco smoking [3].

In both asthma and COPD, inhalation therapy is the recommended gold standard for maintenance treatment [4] as it produces the expected efficacy with lower doses and thus lower occurrence of systemic side effects [5]. Inhalation devices, furthermore, enable patients to achieve the recommended doses with ease [6]. Inadequate (50% or lower) adherence to treatment is a common factor undermining the success of asthma and COPD treatment, leading to the worsening of symptoms, increased morbidity, and increased utilization of healthcare resources [7]. According to international data, 30% of hospital admissions are due to inadequate patient cooperation [8]. In COPD, the incidence of incorrect inhalation technique doubles by the age of 60 years, and quadruples by 80 years [9]. COPD patients have even less adherence to treatment than asthmatics, with estimates of non-compliance ranging from 30% to 70% [10]. Switching to a new inhaler is more difficult for COPD patients than for asthmatics, given that many comorbidities, decreased physical and cognitive abilities impair correct inhaler use and adherence [11].

Currently, there are more than 230 drug-device combinations available for treating respiratory diseases [12]. Innovation was not limited to the design of inhalers [13] or the development of pharmaceuticals [14], since new devices were combined with new drugs discovered and developed to treat asthma and COPD during this period [15]. These include new inhaled corticosteroids (ICSs) and long-acting beta-agonists (LABAs), as well as improved anticholinergic drugs. In addition, the synergistic property of the combination of LABAs and corticosteroids was also recognized, which led to a rapid increase in combination therapies [12]. There are three main areas with expected innovations of inhalation therapy in the future: 1) designing new devices, 2) innovation in formulations and 3) digital technology related to the inhalers [15].

## Smart devices are revolutionizing the treatment of chronic respiratory diseases

Asthma and COPD may have acute worsening periods followed by a complete asymptomatic period, either of temporary or permanent nature [16, 17]. Adjusted medications are based on respiratory function values measured in the office, physical abnormalities at the visit, and the patient’s symptomatic self-assessment, but asthma and COPD symptoms may be exacerbated by certain triggers and symptom-enhancing factors [18]. For a more effective treatment measurements are made regularly, in circumstances of the real lifestyle, if the patient continuously monitors the airway hyperreactivity or inflammation using a home respiratory function device, and records the measured values [16]. The home respiratory function device and the related smartphone application give a sense of security because they can be used to accurately follow-up on the values measured during the office respiratory function test [19]. The measured data displayed by an application can be downloaded to the phone, which also stores the values. In the case of an exacerbation, by reading these, you can know exactly which and how much medicine should be used according to the action plan agreed with your doctor, in order to improve the symptoms. The home respiratory device detects deteriorating values at an early stage. In many cases, at this stage patients do not yet have symptoms [19]. If we see from the measurements that, e.g. pollen concentration, higher air pollution or stress can negatively affect the disease, more severe symptoms can be prevented by intervening in time, modifying the treatment as prescribed, or consulting with the doctor if necessary [19, 20].

**Fig. 1:**
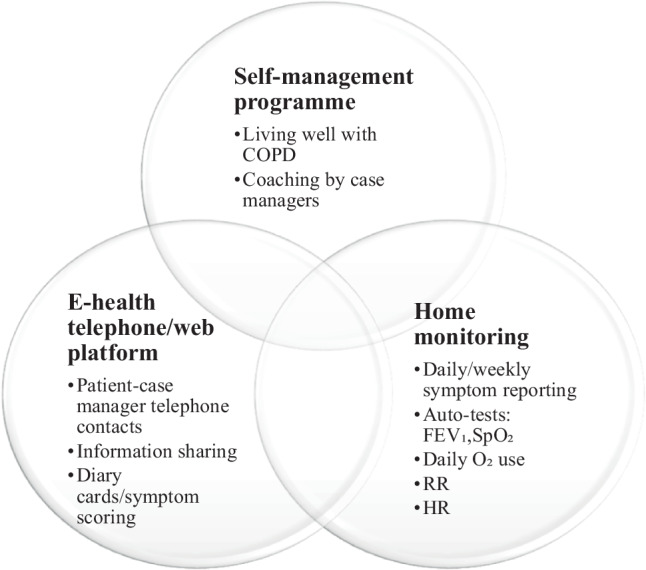
Self-management, home monitoring program in COPD patients. *COPD* chronic obstructive pulmonary disease, *FEV*_*1*_ forced expiratory volume in the first second, *RR* respiration rate, *HR* heart rate, *SpO*_*2*_ blood oxygen saturation levels. (source: www.livingwellwithcopd.com)

New technologies and widespread internet availability provide an opportunity to develop a proper telerehabilitation strategy [16]. Telerehabilitation is also used to treat chronic diseases, including heart disease [21], stroke [22], multiple sclerosis [23], and arthritis [24]. Self-controlled home exercise has been shown to improve respiratory function and health in asthma and COPD [25]. Additionally, home telerehabilitation and home self-management programs have been proven to be as effective as hospital rehabilitation in reduction of acute exacerbations and hospitalizations, and in the risk of emergency department admissions (Fig. 1; [26]).

## New opportunities provided by telemedicine

According to the WHO, telemedicine “includes the provision of health services as defined by information and communication technologies, especially where distance is an obstacle to health care” [27, 28].

Its most important features*:*

- Real-time audio and video communication connecting doctor and patient at the same time (synchronous).
- Storage and transmission of specific health care data (such as findings and recordings, typically as an asynchronous process, e.g. exchange of information does not require the presence of both at the same time).
- Remote monitoring of a specific parameter and the use of various wearable accessories, trackers, applications, sensors and transmission of measured data.
- In a broader sense, this may include telephone or e‑mail counselling, but also counselling on a medical podcast or on question and answer medical sites available on the Internet.

**Fig. 2:**
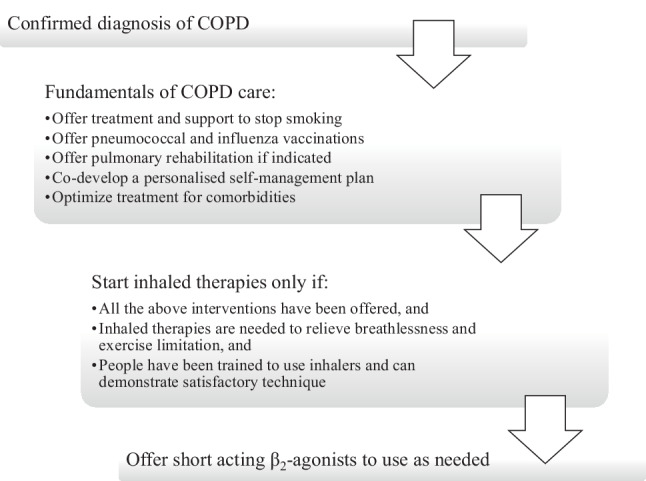
Nonpharmacological management in COPD patients

Based on studies conducted on these topics, both patients and providers are satisfied with the consultation options provided by telemedicine [29]. Options available have been shown to positively influence the patients’ sense of security, health awareness, and adherence to medications in a time-saving and cost-saving manner [30]. Telemedicine, however, should not be considered as a substitute for the wide array of non-pharmacological interventions, such as motivational interview and lifestyle intervention, which are provided at the office with both parties present (Fig. 2).

## Specific telemedicine solutions

- Information through government websites, social media sites, video sharing and popular mobile entertainment applications (e.g. TikTok—ByteDance, Beijing, China).
- Chatbots, applications, solutions based on automatic communication: Chatbots are programs that simulate real online conversation and interaction, which respond to the data provided by the user according to a predefined algorithm. Applications running on mobile devices are capable of alerting the user’s phone using cell information and Bluetooth connections, by comparing a database of certified patients.
- Optional use of sensors and activity trackers: data from built-in sensors (motion sensor, heart rate monitor or pulse oximeter) can help with early diagnosis during individual use, which facilitates the assessment of the condition during the telemedicine visit and later (Table 1).
- Online consultation mainly focuses on controlling symptoms, the classification of the condition based on its severity, and the management of the patient’s pathways in severe cases. In the first phase, the medical staff assess the patient’s condition, give advice on what to do, and, if necessary, refer the patient to the nearest specialist care center [31]. This allows patients to receive care at the appropriate level of progression, saving time and money for the funder without overburdening the healthcare system.

**Table 1:** Wearable biomedical sensors and monitorable factors using sensors

Wearable biomedical sensors	Monitorable factors
Activity trackers	Air quality
Smart watches	Heart rate, heart rate variability
Smart clothing	Respiratory rate, tidal volume
Patches/tattoos	Arterial oxygen saturation
Ingestibles/smart implants	Activity

## Factors influencing the success of telemedicine

The success is influenced by several factors. Access to telemedicine services is significantly lower for lower educated social groups (21%) than in groups with higher education (60%) [32]. The applicability of telemedicine may also be difficult for the aged, especially when combined with geographical barriers (such as small rural settlements) [33]. Numerous studies on telemedicine report on the importance of pre-preparation and the training of patients [34]; however, it is clear that the rapid and hasty introduction of telemedicine also carries a number of dangers [35]. Some of these are technological (whether or not the service is provided on a reliable platform in terms of data security), medical (certain patient problems cannot be treated by telemedicine), while others are legal-ethical (how patients can be transferred to definitive therapy from telemedicine, how to mitigate adverse events and fill in missing patient safety standards) [35]. Another important task is to establish proper quality assurance. Even countries that have been integrating telemedicine into their own healthcare system for more than 15 years (Australia, the USA) need to continually improve the respective regulations [36]. In the United States, telemedicine practice is subject to a license, and in several states, it is subject to passing a professional examination. Defining the legal and ethical framework is also vital, as it is also related to the issue of patient safety and patient path management [37]. International experience shows that important steps have also been taken in the fields of urology [38], dermatology [39], diabetes [40], and pediatrics [41] to make better use of telemedicine.

## Access to digital technology among patients with chronic respiratory disease

Research in the United Kingdom (UK) and Australia has shown that different models of home pulmonary rehabilitation are effective [42, 43]. In a recent Australian study of 254 people, 78% of the sample used a mobile phone and 65% used a computer or tablet regularly [44], 60% of them were happy to participate in and trusted telerehabilitation. Research shows that a large proportion of patients have access to the necessary technology and are using it properly [45, 46].

## Digital technology is the future of pulmonology

The development and routine use of smart inhalers, which collect data and transmit them to the patient’s general practitioner (GP), allow symptoms to be monitored over time. In 2019, the Food and Drug Administration (FDA) approved the first smart inhaler, which measures and evaluates the respiratory function [47]. Asthma requires measuring lung capacity using flow meters, which is why Health Care Originals has developed a portable device to measure cough, breathing, heart rate, temperature and other data [48]. The device warns the patient and/or the doctor if any of the parameters deviate from the normal values and warns the patients to take their medication regularly. The data can be shared by the pulmonologist and GP, making it easier for them to treat asthma patients. It is essential to check the correctness of the medication dosing technique, and it is also important that the patient follows the doctor’s instructions. Propeller Health [49], Cohero Health [50], and Amiko [51] have also developed smart inhalers, and the National Health Service (NHS)-recommended myCOPD [52] app is also helping patients monitor their medication.

In the future, other connected sensors placed in clothing are expected to measure exhaled nitric oxide fraction, physical activity, or environmental pollution, and monitor physiological parameters [53]. Based on patents approved by Samsung in February 2019, the company started developing a smart shirt that monitors lung activity. The shirt is connected via sensor to the patient’s smartphone, and can therefore diagnose asthma, pneumonia, bronchitis, or COPD. For proper diagnosis, the wearers’ gender, age, weight, and height, as well as their medical history must also be taken in account [54].

According to some experts, the next step is to incorporate various sensors into clothing that can show if the patients are exposed to too many harmful chemicals or too much smoke or air pollutants in their environment [55]. Based on the results, the tool provides advice, which can vary between preventive and emergency measures. The Spire and the Vitali Smart Bra & Gem smart bras, for example, are wearable devices that use motion sensors to measure patient activity and respiration [54]. Although manufacturers do not fully disclose their measurement principles, such systems can analyze the respiratory function, providing biofeedback to the patient.

The idea of applying these new technologies in pulmonary rehabilitation is very exciting; however, future research should explore the efficiency of such innovative methods comparing them with traditional model of pulmonary rehabilitation. A key factor in pulmonary rehabilitation programs may be the value of enjoyment. The impact of leisure-focused work-outs, such as tai chi, yoga, Nordic walking, and dancing may be key to enticing people who are not interested in participating in traditional gym-based exercise [56].

Pulmonary rehabilitation, even if provided remotely, must retain its cornerstones, such as education and motivation, which are most successful when conducted in person [57]. Moreover, the selection of patients suited for telerehabilitation, the measurement of certain outcome variables, the development of emergency and intervention plans require also the personal presence of patients and physician [58]. The WHO renewed recommendations include detailed suggestions for the management of COPD patients, which are also reflected in everyday therapy [59]. To facilitate this, programs are needed to motivate patients to improve their own condition and increase health literacy about the disease. Evidence shows that a well-informed patient is more likely to adhere to treatment [60]. COPD has a significant impact on patients’ quality of life, unfortunately it is usually only recognized in moderate to severe cases, when it is already severely limiting [61], and is often associated with anxiety, depression, sleep disturbances and cognitive decline [62]. The lack of knowledge has been shown to compromise the effectiveness of the treatment and may exacerbate the disease, therefore education programs, improved doctor-patient communication and patient education tools (forms, emails); and the use of self-monitoring methods (medication diaries, mobile apps) are of utmost importance, because education and being well-informed positively influence doctor-patient collaboration and ultimately the outcome of this chronic disease [63].

## Conclusion

In order to unify pulmonary telerehabilitation, a joint effort of the scientific and professional society is needed for international organizations. This effort should be developed in collaboration with the most prominent international companies in the field of pulmonology, the European Respiratory Society (ERS) and the American Thoracic Society (ATS). Priority will be assigned to the protection of personal rights, regulating access to health data and preventing manipulation, and the security of the transmission, conversion and storage of health data, i.e. cyber security. The conclusion of our study is that using digital devices may improve the adherence to medication of patients with chronic respiratory diseases. Thanks to the use of algorithms and the analysis of real-time data, it is possible to detect symptoms in the early exacerbation phase and by altering the therapy in time, we can prevent deterioration. Given the accuracy, predictability, and personalization that characterizes digital remote monitoring devices, it is imperative that current and future technologies be included in the treatment of patients with chronic respiratory diseases.

## Abbreviations

ADAMM: Automated device for asthma monitoring and management
ATS: American Thoracic Society
COPD: Chronic obstructive pulmonary disease
ERS: European Respiratory Society
FDA: Food and Drug Administration
GP: General practitioner
ICS: Inhaled corticosteroids
LABA: Long-acting bronchodilators
LAMA: Long-acting muscarinic antagonist
NHS: National Health Service
PR: Pulmonary rehabilitation
UK: United Kingdom
WHO: World Health Organization
